# Toxicology of Heavy Metals to Subsurface Lithofacies and Drillers during Drilling of Hydrocarbon Wells

**DOI:** 10.1038/s41598-020-63107-3

**Published:** 2020-04-09

**Authors:** Emmanuel E. Okoro, Amarachi G. Okolie, Samuel E. Sanni, Maxwell Omeje

**Affiliations:** 1 Petroleum Engineering Department, Covenant University, Ota, Nigeria; 2 Chemical Engineering Departement, Covenant University, Ota, Nigeria; 3 Physics Departement, Covenant University, Ota, Nigeria

**Keywords:** Environmental sciences, Health occupations, Risk factors

## Abstract

This study investigates the toxicological effects of heavy metals on lithofacies of the subsurface in a drilled hydrocarbon well as well as, to the drilling crew and people in an environment. The pollution levels of selected heavy metals were considered alongside their ecological effects during dry and wet seasons. The health hazard potential of human exposures to the metals, were estimated in terms of intensity and time using the USEPA recommended model. The heavy metal concentration for each layer decreased across the lithofacies as follows; Layer 5> Layer 4> Layer 3> Layer 2> Layer 1. The average concentrations of the heavy metals present in the samples obtained from the formation zone, varied significantly and decreased in the order of Al> Zn> Ni> Pb> Cr> Cu> Cd> As> Hg. The highest concentration of Al, Cu, and Zn in this present study were within the maximum allowable limits whereas, those of As, Cd, Hg and Ni were all above their maximum allowable limits. Among the transition metals analysed, the maximum mean daily dose of Pb (9.18 × 10^−6^ mg/kg/d) and Cr (1.42 × 10^−6^ mg/kg/d) were confirmed susceptible to human carcinogens and environmental toxins. The estimated hazard quotient shows that the dermal pathway is the most likely route via which the drilling crew and people in the environment can get contaminated. The cancer risk values for the Pb (7.72 × 10^−4^), Cd (1.35 × 10^−1^), Ni (9.97 × 10^−3^), As (1.50 × 10^−1^) and Cr (3.16 × 10^−3^) are all above the acceptable values. The cancer risk contribution for each metal was in the order of As> Cd> Ni> Cr> Pb. Layer 5 had the maximum Geo-accumulation index for the heavy metals considered. This higher Geo-accumulation index noted at the depth in Layer 5 may be attributed to the effect of water basin with turbidity currents, deltas, and shallow marine sediment deposits with storm impacted conditions. Also, the pollution from lead (Pb) in the dry season was maximum with an I_geo_ value> 5 for all the lithofacies considered because of the low background concentration of the metal. During the wet season, the heavy metal pollution rate was moderate for Zn whereas, it was extremely polluted with respect to Pb. The ecological risk potential of Pb shows that the associated ecological risks range from 536 – 664 in the wet season (i.e. extremely strong) and 2810 – 3480 in dry season (extremely strong). The high level of Pb pollution found in the area at such shallow depth may be due to the sedimentary folds possibly caused by the full spectrum of metamorphic rocks and primary flow structures at shallow depths. This was used to identify the environmental sensitivities of the heavy metals during the dry and wet seasons.

## Introduction

Hydrocarbon reservoirs can only be accessed through drilling of the subsurface rocks/ formations. Rotary drilling technique has been currently employed in drilling of hydrocarbon wells which run into thousands of feet below the ground surface^1^. A hydrocarbon well is drilled by rotating a drill-bit attached to the lower end of the drill-string (drill-pipe). Cuttings which are the true representations of subsurface lithofacies are being generated during drilling, and are removed by continuous circulation of the drilling fluid in the annular space between the wellbore and the drill string^2,3^. At the surface, settling pits and mechanical equipment (shale shaker) extract the cuttings (pieces of lithofacies drilled), and allow the clean drilling fluid to be re-circulated downhole in the closed loop drilling setup.

The presence of heavy metals is a common occurrence in the subsurface formation layers, and as such, handling of the metal wastes generated during drilling has resulted in human exposures. Heavy metals as implied in this study, are classified as toxic metals, irrespective of their atomic densities or masses^4^. Davies et al.^5^ made reference to the relationship between health and the environment; they stated that health cases are often related to the occupational environment. Health problems resulting from the presence of heavy metals and their toxicity have been revealed in many parts of the world^6–8^. Due to the nature of drilling programmes and the location of hydrocarbon wells, drilling crews often work on the rig and sleep on the platforms which serve to accommodate them in the environments until their shifts are over. The science of toxicology shows that these workers can have some health challenges due to exposure to heavy metals/ elements in the natural environment (particularly rocks or mined mineral resources). Heavy metals have drawn considerable attention due to their non-biodegradable nature and toxicities^9^. There is also evidence that high exposure to low doses of cancer-causing heavy metals may cause several types of cancers^10^. Gbadamosi *et al*.^9^ highlighted the increased lifetime risks of lung cancer resulting from occupational exposure to dusts and mists containing hexavalent chromium.

The ecological effects of heavy metals in soils are closely related to their contents and distribution of species in the solid and liquid phases of soils. Literature results indicate that the adsorption of heavy metals on soil particles is not only restricted to the formation of surface complexes but can also take place in the structure of minerals^11^. According to Wuana and Okieimen^12^, heavy metals occur naturally in the soil environment from the pedogenetic processes of weathering of parent materials at levels that are regarded as trace (i.e. <1000 mgkg^−1^) despite being rarely toxic. The most common heavy metals found at contaminated sites in order of abundance are Pb, Cr, As, Zn, Cd, Cu, and Hg^13^. The specific type of metal contamination found in a contaminated soil is directly related to the operation that occurred at the site. The range of contaminant concentrations and the physical and chemical forms of contaminants will also depend on activities and disposal patterns. Soils may contain metals in solid, gaseous, or liquid phases, and this may complicate the analysis and interpretation of reported results. For example, the most common method for determining the concentration of metal contaminants in soils is “total elemental analysis” (USEPA Method 3050). Literature has soil concentration ranges and regulatory guidelines for some heavy metals^14–16^. For oil and gas operations in Nigeria, the Department of Petroleum Resources has recommended guidelines on remediation of contaminated lands based on two parameters namely, intervention and target values. The intervention values indicate the quality or functionality as it relates to soil, human, animal, and plant life or how threatened they are being seriously impaired. Concentrations in excess of the intervention values correspond to serious contaminations. Target values indicate the soil quality required for sustainability and are expressed in terms of the dictates of the remedial policy while the target values indicate the soil quality levels ultimately aimed at.

Heavy metals are considered serious pollutants not only because of their persistence and non-degradability in the environment but also because most of them have toxic effects on living organisms when they exceed a certain concentration^17^. Therefore, there is need to identify and estimate the key potential impact of the heavy metals present in these different lithofacies encountered during drilling of hydrocarbon wells at depths greater than 10000 ft, because, the drilling activities will have several interactions with the environment. This helps to quantify the risks associated with their exposures. Exposure is the contact of a contaminant with a receptor, and the health hazard of the exposure in terms of intensity and time was estimated using the USEPA recommended model. These above mentioned hazard implications have not previously been considered in the study area. Risk assessments help define accurate hazard potentials as well as identify the lithofacies with high health and environmental risks. The aim of this study is to examine the presence and pollution levels of selected heavy metals in different subsurface lithofacies in order to determine and evaluate the potential toxicity impacts of their exposures, as well as identify the environmental sensitivities which help to ascertain their ecological impacts during wet and dry seasons within the study area.

## Materials and Methods

### Geographical location of the study area

**Figure 1:**
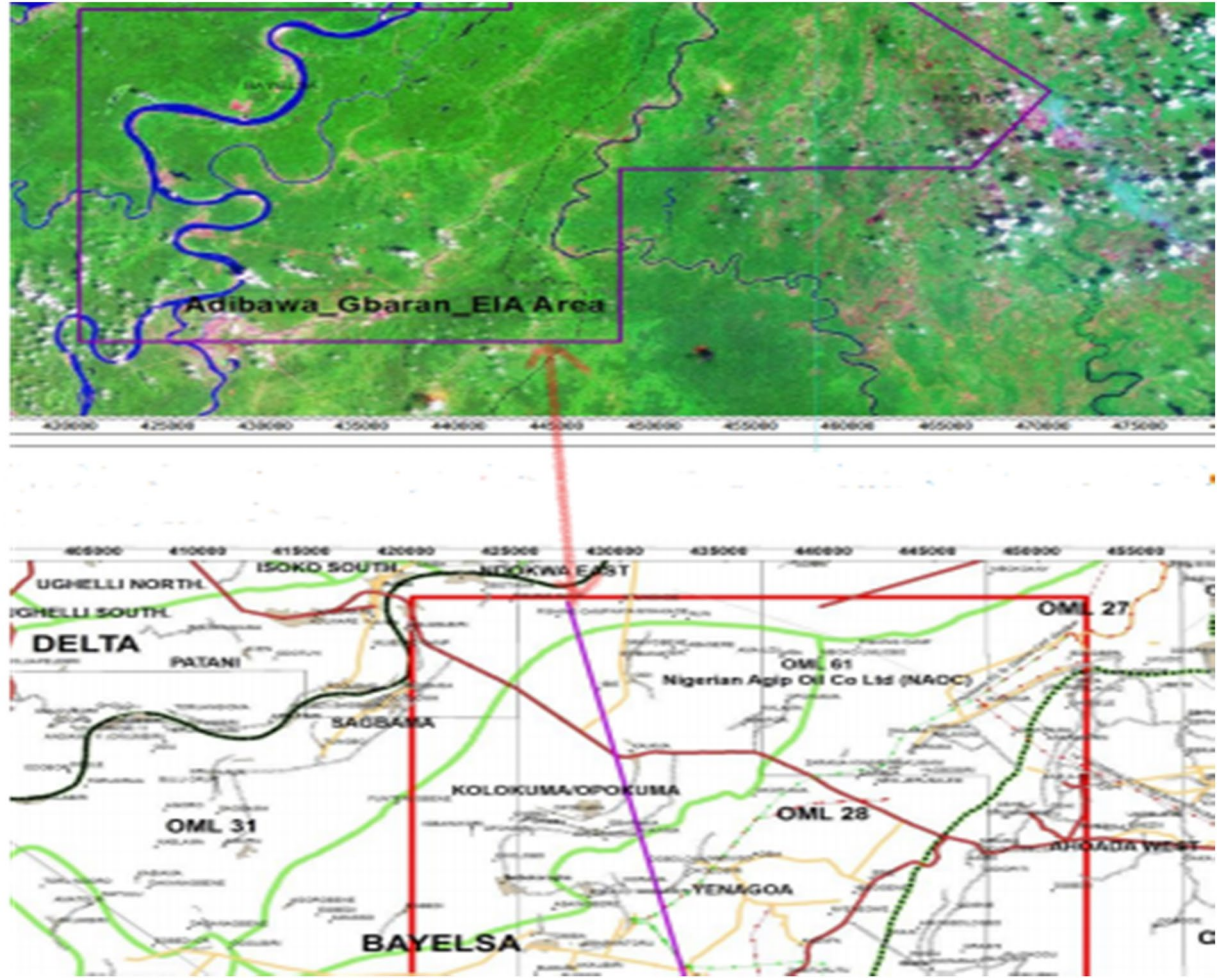
Map of the Study Area^35^.

The study area is known to have a vast deposit of hydrocarbons (crude oil and gas). It is located in Bayelsa State, about 110 km North-West of Port Harcourt and about 27 km South-West of Yenagoa (the state-capital of Bayelsa) as shown in the map of the study area (Fig. 1). The area experiences humid tropical climate which is characterized by wet and semi-hot equatorial regimes. There is a high relative humidity all year round and during the dry season, it ranges from 73 to 80%, while in the wet season it ranges from 82 to 90%. The maximum temperature ranges from 32 to 34 °C (in the dry season), while the minimum temperature ranges from 28 to 32 °C in the wet season. The vegetation is characterized by the tropical fresh water swamp forest, with perennial heavy rain falls, unstable marshy terrain and seasonal flooding.

### Geology of the study area

**Figure 2:**
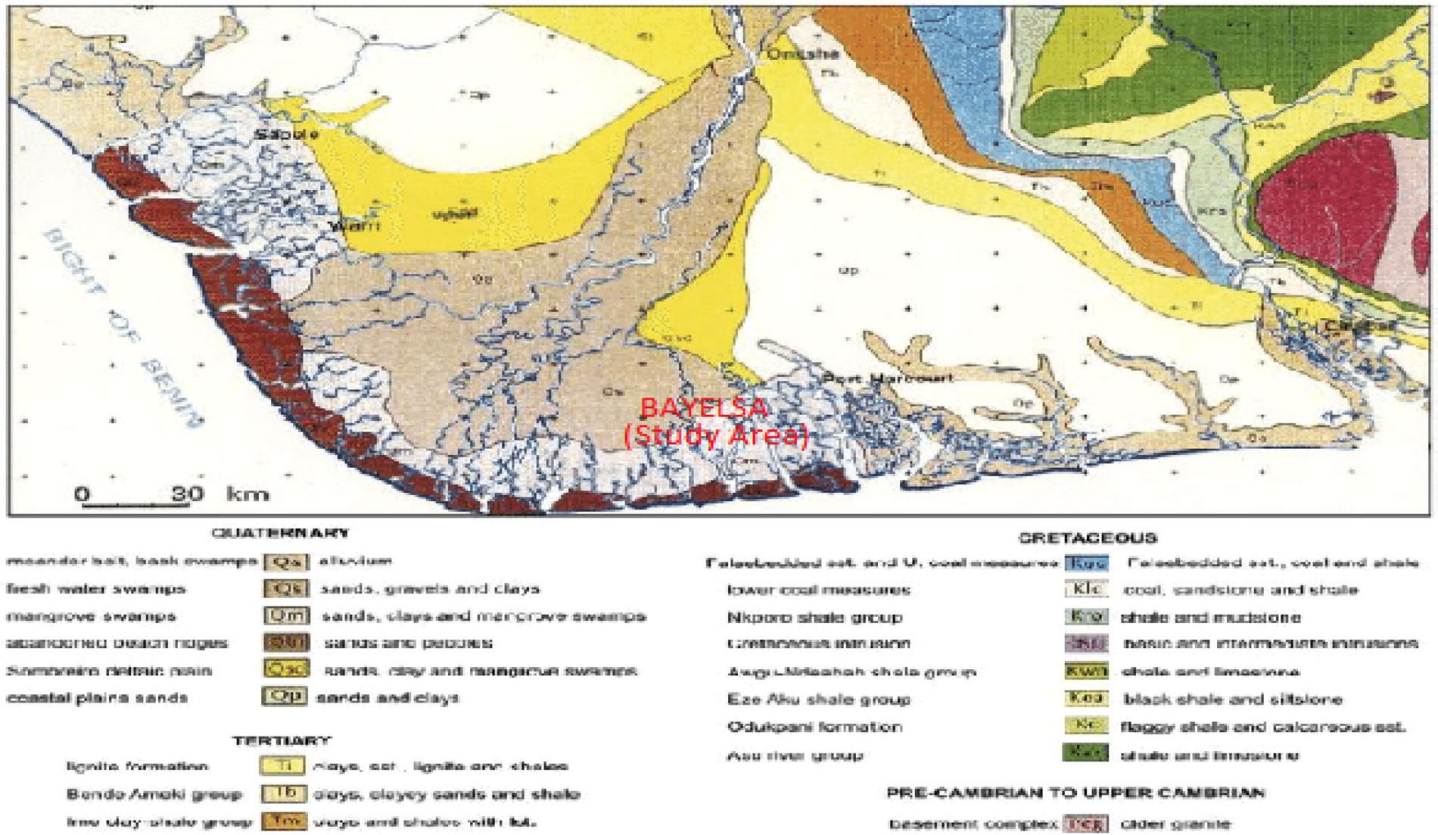
Geologic Map of the Study Area^19^.

The Niger Delta is the Cenozoic gross offlap clastic succession built out on top of the Anambra Basin. The delta was formed as a major continental embankment that spreads out over the cooling and subsiding oceanic crusts generated by the separated African and the South American lithospheric plates^18,19^. The litho-stratigraphic sequence of the subsurface units comprise majorly of transgressive marine akata shales, the petroliferous paralic agbada formation, and the continental Benin Sands. Oil reserves will be about 40 billion barrels, with gas reserves close to 40 trillion cubic feet^20^. The existing traps are mainly deep closures (rollover anticlines in growth faults) containing relatively rare stratigraphic traps. Hydrocarbon is sourced from the marine shales with land plant materials transforming mainly into types 3 and 2/3 organic matters within the oil window ranging from 9000 to 14000 feet-deep^21^. The reservoir is manly characterized by a shore, a beach, channel sand and occasional turbidity^22^. The crude oil in Niger Delta has low amounts of sulfur, nickel and wax and is non-graded. The geology of the study area is as illustrated in Fig. 2.

### Materials and method for toxic metal contents in the samples

In this study, samples of lithofacies of different drilled subsurface layers were collected from an oil and gas field. A total of five (5) samples of different subsurface layers were collected during drilling for investigation. They were crushed, pulverized and sieved using a 75 µm mesh for homogeneity. Thereafter, the crushed materials were put in a plastic vial and labelled with a marker for easy identification before sending them to the Bureau Veritas Laboratory Ltd, Canada, for analysis. About 0.2 g of each sample was accurately weighed into a container perfluoroalkoxy polymer, which was then placed in a microwave pressure vessel (Ethos Plus Microwave Lactation, Milestone Inc., Shelton, CT, USA), using the 3052 method contained in the US EPA standard^23^. After the addition of 4 ml concentrated nitric acid and 0.5 ml concentrated hydrochloric acid, the samples were digested in a microwave whose power was progressively increased to 400 W in 40 min. After cooling, the solutions were accurately diluted using 100 ml water^24^. However, an open digestion in a glass beaker was conducted by measuring and heating 0.5 g portion of the mixture with 12 ml of aqua regia for 40 mins, followed by evaporation to dryness. 25 ml of concentrated hydrochloric acid and 2.5 ml of hydrogen peroxide were added to the hot residue with an accurate dilution of 50 ml of water. One replicate per digestion method was done for each sample. The total content of heavy metals in the building materials was analysed using ICP-MS instrument connected to the intuitive WinLab32 software system which comprises of tools which help to analyse, report as well as generate data. To calibrate the equipment, standard solutions (panreac) of 100 mg/l of all metals was used and as such, the solutions were calibrated from 10 – 100 ppb.

### Quality control for the analysis of toxic metals in the formation samples

In this study, the quality control for the analysis of the samples using Perkin Elmer ICP-MS was conducted using standard operating procedures (SOPs) specified in the manufacturer’s manual. All the equipment used in this study were calibrated before taking measurements. A calibration curve was obtained for ICP-MS before the analysis was conducted in order to ensure accuracy. The analytical method was assessed by analysing the US EPA^23^ 3052 standard reference material.

### Formation samples x-ray diffraction analysis

The samples for X-Ray diffraction analysis were sent to the Nigerian Geological Research Laboratory (NGRL) in Kaduna, Kaduna State. The NGRL Kaduna uses the Schimadzu 6000 model. The prepared powder sample is known as the Bulk Sample. The sample was spread lightly on the sample holder made of aluminium material by using a smooth slide. The bulk sample range of 2° to 60° theta was set and the running rate was set to 6° per minute. After which the sample holder was carefully placed on the loading point of the movable Goniometer arm that contains a clamp capable of gripping the sample firmly^25^. 10 g of the powder sample was placed inside a neat test tube with the assistance of a spatula. Distilled water was then added to dissolve the sample and the mixture was later placed in a centrifuge and allowed to spin at 5 rpm for 5 minutes. Thereafter, the sample was taken out and the floating materials were decanted. Distilled water was added, the mixture was mixed thoroughly and placed on the centrifuge for the second time. The process was repeated about 5 times since the mixing was dependent on the rate at which individual sample become a clear suspension. 3-5 drops of 0.6% sodium hexameter phosphate solution was added which led to the formation of a clear suspension above the test tube; below the test tube were other unwanted deposits. Some quantities of the suspended clay that was formed above the test tube was taken with a dropper and placed on a well labelled clean glass slide. This was allowed to dry for 24 hours.

### Hazard quotient (HQ) and hazard index (HI)

HQ is a unitless number that shows the probability of a drilling crew member or people in the environment will suffer an adverse effect from heavy metal exposure. HQ calculation is used to quantify the non-carcinogenic health risk.

1 $$HQ=\frac{ADD}{{R}_{f}D}$$

Where the *R*_*f*_*D* values were adopted from US EPA^26^ risk-based concentration table, and the ADD is the acceptable oral dose for a heavy metal.

### Carcinogenic risks assessment

The cancer risk indices for the drilling crew and people in the environment were estimated for the two pathways. Carcinogenic risk is the incremental probability to develop cancer over a lifetime as a result of exposure to these toxic heavy metals. Equation (2) was adopted in estimating the lifetime cancer risk for each heavy metal through the two pathways.

2 $$Ris{k}_{pathway}=\mathop{\sum }\limits_{k=1}^{n}AD{D}_{k}CS{F}_{K}$$

Where Risk is a unitless probability of an individual developing cancer over their lifetime. *ADD*_*k*_ (mg/kg/day) and *CSF*_*K*_ (mg/kg/day)^−1^ are average daily dose and cancer slope factors for each heavy metal respectively.

### Geo-accumulation index

Equation (3) was used to estimate the degree of heavy metal pollution in the soil around the study area. The classification used to estimate the pollution level of the calculated Geo-accumulation index (I_geo_) is: I_geo_ value ≤ 0 (unpolluted), I_geo_ values between 0 and 1(unpolluted-moderately polluted), I_geo_ values between 1 and 2 (moderately polluted), I_geo_ values between 2 and 3 (moderately-strongly polluted), I_geo_ value between 3 and 4 (strongly polluted), I_geo_ value between

4 and 5 (strongly-extremely polluted), I_geo_ value> 5 (extremely pollution)^27^.

3 $${I}_{geo}=lo{g}_{2}\frac{{C}_{n}}{1.5{B}_{n}}$$

Where, C = contaminant concentration of the heavy metals, and BV = background value of the heavy metals.

### Contamination factor

The present study also considered the overall contamination level of the study area by each of the heavy metals; the contamination factor (CF)^28,29^ was adopted here. It is the ratio of the measured heavy metal concentration to the background values as presented in Eq. (4).

4 $$CF=\frac{{({C}_{metal})}_{sample}}{{({C}_{metal})}_{background}}$$

### Ecological risk potential

The ecological risk assessment was estimated to evaluate ecological effects of the heavy metals transported from different subsurface lithofacies during drilling of hydrocarbon wells in the area of study. According to European Environment Agency and United States Environmental Protection Agency, the released heavy metals in the environment during drilling has effect on all living organisms in the variety of ecosystems which make up the environment. Ecological risk assessments are based on scientific data, and Eqs. (5) and (6) were adopted in estimating the likelihood that these heavy metals will pollute the ecosystem of the study area. These equations estimate the average potential ecological risk index of each heavy metal and the comprehensive potential ecological risk index respectively.

5 $${E}_{R}^{i}={T}_{R}^{i}\times {C}_{F}^{i}$$

6 $$RI=\mathop{\sum }\limits_{i-1}^{m}{E}_{R}^{i}$$

Where, ${E}_{R}^{i}$ is the potential ecological risk index of each heavy metal, ${C}_{F}^{i}$ is the contamination factor of each heavy metal, ${T}_{R}^{i}$ is the biological toxic factor of each heavy metal (the values adopted for this study are: Zn = 1, Cr = 2, Ni = 5, Pb = 5), and RI is the comprehensive potential ecological risk index.

## Results and Discussions

### Heavy metal concentration

There are concerns on environmental levels of heavy metals and their effects on human health^5^. Table 1 shows the concentrations of the nine (9) heavy metals considered in this study, and their distribution in each lithofacies collected from the study area. The Excel work sheet for the heavy metals uncertainty calculation is attached as supplementary document.

**Table 1:** Heavy metals distribution over the lithofacies of the subsurface considered.

Heavy Metal	Subsurface Lithofacies				
	L1	L2	L3	L4	L5
Lead (mgKg^−1^)	163 ± 0.10	174 ± 0.10	185 ± 0.15	192 ± 0.10	202 ± 0.15
Cadmium (mgKg^−1^)	16 ± 0.10	20 ± 0.10	24 ± 0.15	28 ± 0.20	31 ± 0.10
Mercury (mgKg^−1^)	12 ± 0.15	14 ± 0.10	15 ± 0.15	18 ± 0.10	19 ± 0.20
Copper (mgKg^−1^)	26 ± 0.10	29 ± 0.15	32 ± 0.10	34 ± 0.05	35 ± 0.10
Arsenic (mgKg^−1^)	14 ± 0.10	16 ± 0.15	19 ± 0.15	22 ± 0.15	25 ± 0.10
Chromium (mgKg^−1^)	74 ± 0.15	77 ± 0.10	82 ± 0.15	90 ± 0.15	95 ± 0.05
Zinc (mgKg^−1^)	227 ± 0.15	238 ± 0.10	252 ± 0.05	264 ± 0.10	272 ± 0.10
Nickel (mgKg^−1^)	187 ± 0.10	198 ± 0.15	212 ± 0.10	226 ± 0.15	232 ± 0.10
Aluminium (mgKg^−1^)	457 ± 0.15	490 ± 0.10	534 ± 0.20	587 ± 0.10	606 ± 0.15

It was deduced from Table 1 that the mean concentrations of the heavy metals generally varied from 12 – 606 mg/kg for all lithofacies. The nine ((9) heavy metals were found in the six (6) layers of the lithofacies under consideration. Layer 5 (9,050 – 9,590 ft) had the highest heavy metal concentration for all the nine (9) heavy metals considered in this study. The heavy metal concentration for each layer decreases across the lithofacies as follows; Layer 5> Layer 4> Layer 3> Layer 2> Layer 1. The heavy metal distribution had a uniform increment from Layer 1 which is the topmost formation from which the samples were collected up to the 5^th^ Layer (i.e. Layer 5). The average concentration of the heavy metals found in the formation samples varied significantly and decreased in the order of Al> Zn> Ni> Pb> Cr> Cu> Cd> As> Hg. The average range for individual heavy metal is as follows: Al (457 – 606 mg/kg), Zn (227 – 272 mg/kg), Ni (187 – 232 mg/kg), Pb (163 – 202 mg/kg), Cr (74 – 95 mg/kg), Cu (26 – 35 mg/kg), Cd (16 – 31 mg/kg), As (14 – 25 mg/kg), and Hg (12 – 19 mg/kg). Pb is a toxic metal often studied in literature, and it has a wide range of biological effects depending on the level and duration of the exposure^30^. An attempt was made to compare these heavy metal concentrations with international standards (WHO and USEPA), since each country and region has varying maximum allowable limits (standards) for the heavy metals. The highest concentration of Al, Cu, and Zn in this present study were within the maximum allowable limits according to US EPA^24^ but, the high concentration values of As, Cd, Hg and Ni in this study were all above the maximum allowable limits of 20, 3, 30 and 72 mg/kg respectively^24^ hence, an implication for hazard.

**Figure 3:**
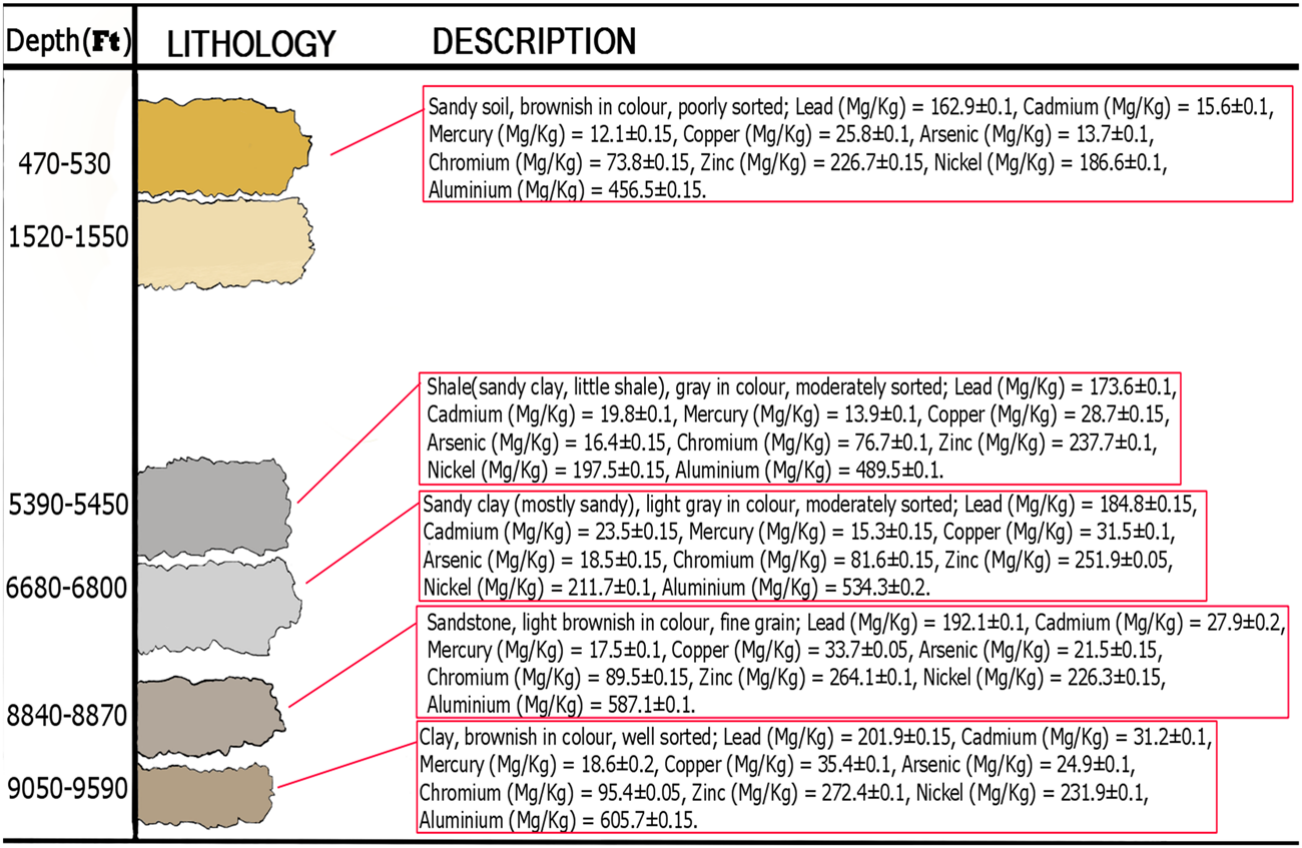
Heavy Metals distributions across the Subsurface Lithofacies considered.

The highest value of chromium (Cr) obtained in this study is 95.4 mg/kg, while the WHO/IPCS^31^ and US EPA^32^ maximum allowable limit is 85 mg/kg. This value which is higher than the maximum allowable standard can pose a health risk to both the drilling crew and the people in the environment. IARC^33^ concluded in their study that there was sufficient evidence to classify Cr compound as a Group I human carcinogen. US EPA^10^ placed the maximum allowable limit of lead (Pb) in soil to be in the range of 180 − 200 mg/kg; but the maximum concentration of Pb in the lithofacies under investigation is 202 mg/kg for Layer 5; this is slightly above the acceptable range and could pose some health concerns. Figure 3 represents the measured heavy metal concentrations for each layer of the subsurface lithofacies. Comparing the analysed lithofacies with target values for heavy metals set by Department of Petroleum Resources (DPR) in Nigeria for standard soil, it was observed that the heavy metals values of Pb, Cu, Cr and Ni in the five lithofacies were greater than the DPR target values of 35 mg/kg, 0.30 mg/kg, 20 mg/kg, and 140 mg/kg respectively for these particular heavy metals^12^. Exposure to these Pb concentrations which are about 4 times the target value, can result in a wide range of biological effects depending on the level and duration of exposure. Pb performs no known essential function in the human body. Cu is essential but the high dose (more than 9 times the target value) can cause anaemia and intestinal irritation to the people in direct contact with these cuttings from the lithofacies. Cr mobility depends on sorption characteristics of the lithofacies and the amount of organic matter present; this trend was observed as the Cr concentration exceeds the target values and increased as the depth increases. Ni on the other hand is essential in small doses, but it can be dangerous when the maximum tolerable amounts are exceeded. This was the case in this study as the Ni concentrations for the different lithofacies were greater than the target values set by DPR.

**Figure 4:**
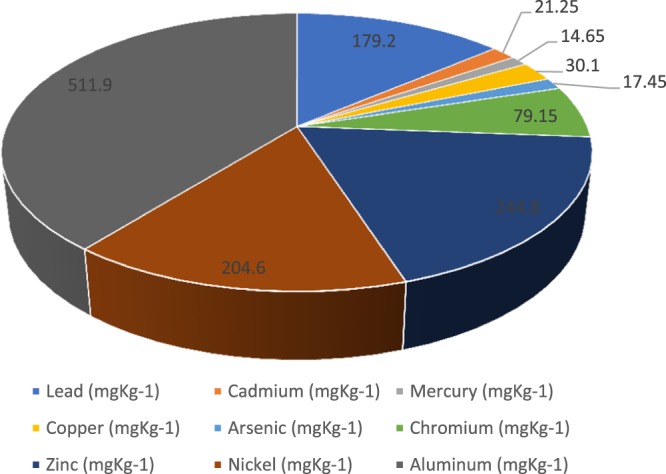
Mean concentration of the Heavy Metals present in the Lithofacies investigated.

The range of contaminant concentrations and the physical and chemical forms will also depend on activities and disposal patterns for the contaminated waste on the site. The transportation of these lithofacies and these heavy metals from the subsurface to the surface environment can lead to adverse impacts. Drilling operation is thus, one of the major ways human activities can contribute to environmental pollution through surface exposure to these naturally occurring subsurface heavy metals of significant concentration. The mean concentration of Aluminium (512 mg/kg), Zinc (245 mg/kg), Nickel (205 mg/kg), and Lead (179 mg/kg) are significant when compared to five (5) heavy metals under investigation (Fig. 4). The mean concentration values of Pb and Cu in the lithofacies considered are within the intervention heavy metal limits set by DPR.

### Heavy metal health risk assessment in the lithofacies

An assessment of soil pollution by heavy metals involves sampling and analysis of representative lithofacies and comparing the soil contamination/pollution rates with regulatory standards. US EPA^34^ standard was adopted in calculating the average daily dose, hazard quotient and index for non-carcinogenic/carcinogenic risk indices of the heavy metals under investigation. The severity of possible adverse health effects is related to the type of heavy metal and its chemical composition, and is also time- and dose-dependent. Table 2 shows the estimated parameters for annual daily dose risk assessment evaluation.

**Table 2:** Parameter for Annual Daily Dose computation.

Parameter	Value	Reference
Daily Consumption (IR) (kg/person/day)	0.38	US EPA (2007)
Exposure frequency (EF) (day/year)	28	Site investigation
Exposure duration (ED) (year)	20	Site investigation
Body weight (BW) (kg)	72	US EPA (2000)
Mean exposure time (AT) (year)	64.95	Yang et al., (2018)
Concentration of each heavy metals (C) (mg/kg)	—	Laboratory

**Figure 5:**
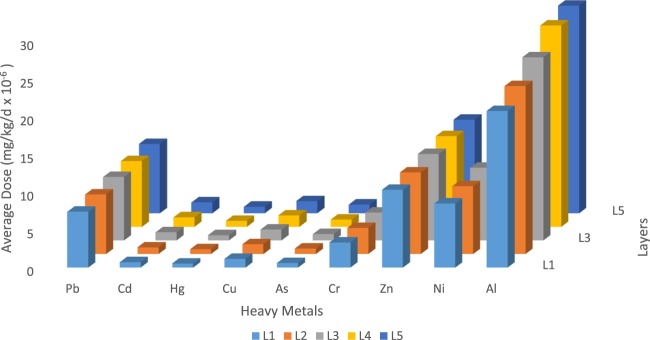
Average Daily Dose intake of the Heavy Metals.

Figure 5 shows the average daily exposure of heavy metals to the drilling crew and people in the environment and the equation used for the computation was adopted from^23,35^. This gave a comprehensive understanding of the heavy metals’ contamination in the lithofacies. Both the mechanisms of toxicity and the critical effects may vary with the type of metal. Additionally, short-term exposures may produce target organ effects which may be very different from those produced by a similar dose over a longer period of time. This section accounts for the most significant contaminant and illustrates the severity of this pressing environmental problem.

Among the transition metals accepted as human carcinogens in one form or another^33^, the maximum mean daily dose of Pb (9.18 × 10^−6^ mg/kg/d) and Cr (1.42 × 10^−6^ mg/kg/d) can be a concern for the drilling crew and environment^34^. The average daily dose of Pb and Cr increased deeper at the subsurface. According to the US EPA Integrated Risk Information System statistics, 3 − 3.5 × 10^−3^ mg/kg per day is the recommended reference dose range for oral intake for Pb and Cr respectively. The effect of these two heavy metals were predominantly located in Layer 5, because their concentrations increased with depth. Also Layer 5 is predominately clay formation and this observation is in line with other literature which have shown the affinity of these heavy metals with the physical and chemical characteristics of the lithofacies^36,37^.

The available reference doses (R_F_D) in mg/kg/day and cancer slope factors (CFS) in US EPA^32^ were used for non-carcinogenic and carcinogenic risk assessments of the heavy metals.

### Estimation of hazard quotient (HQ) and hazard index (HI)in the study area

**Figure 6:**
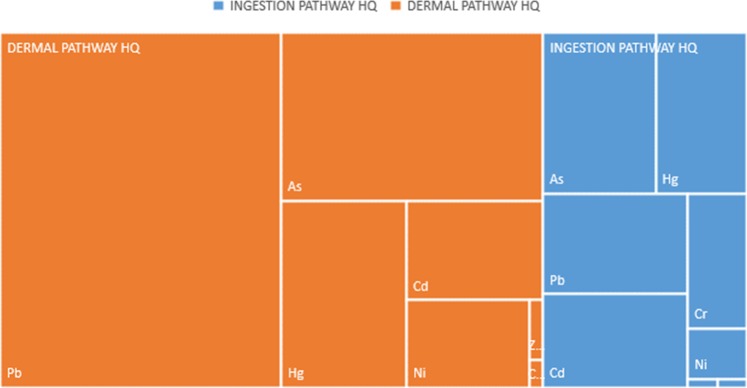
Hierarchical representation of the Ingestion and Dermal Pathways Hazard Quotient.

The hazard index (HI) for the non-carcinogenic risk, is the summation of the heavy metals calculated hazard quotient. If HI < 1, it can be assumed to reflect safety, whereas HI > 1 is assumed to indicate potential for carcinogenic effects. Figure 6 shows the non-carcinogenic risk through ingestion pathway for the heavy metals in each lithofacies layer. The hazard quotient of the heavy metals decreased in this order; Pb> As> Hg> Cd> Cr> Ni> Cu> Zn; and the same trend was observed for the different lithofacies collected for analysis. The computed values are detailed in the Excel work sheet is as attached in the supplementary document. The hazard index value for the ingestion pathway (0.058) and dermal pathway (0.153) considered were less than 1; thus, there is no obvious immediate risk through this pathway to the drilling crew and the people in the environment (Fig. 6). But, the dermal pathway is the most likely route via which the drilling crew and people in the environment can get contaminated.

### Estimated carcinogenic risk assessments

**Figure 7:**
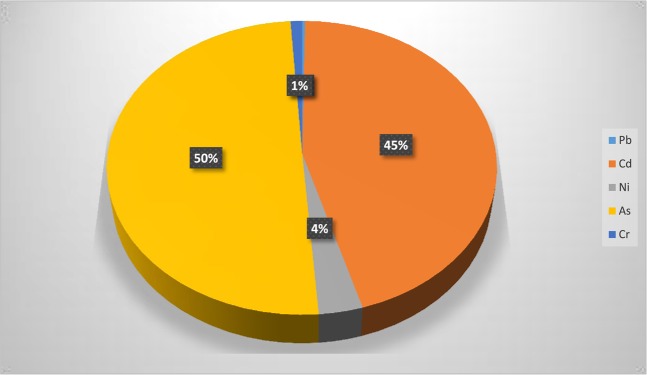
Carcinogenic Risk for five of the Heavy Metals identified in the Lithofacies.

The carcinogenic risk was calculated using data obtained for Pb, Cd, Ni, As and Cr. Figure 6 shows that As (50%) and Cd (45%) are the highest contributors to cancer risk. Cancer risk within the range of 1 × 10^−6^ to 1 × 10^−4^ is considered acceptable by US EPA. The cancer risk values for the Pb (7.72 × 10^−4^), Cd (1.35 × 10^−1^), Ni (9.97 × 10^−3^), As (1.50 × 10^−1^) and Cr (3.16 × 10^−3^) are all above the acceptable values. The cancer risk contribution for each heavy metal decreased in this order: As> Cd> Ni> Cr> Pb (Fig. 7), and the concentrations of the heavy metals varied with the depth and lithofacies. The lithofacies showed that the heavy metal concentration in Layer 5 (clay formation) were higher than in other layers sampled in this study. In this area of study, the drilling crew and people in the environment are at risk. The dermal pathway seems to be the major route to excess lifetime cancer risk followed by the ingestion route.

### Lithofacies heavy metals pollution assessment

The results of some heavy metals within the study area are summarized in Table 3^25^. This background information in the study area were further used for pollution assessment analysis in this study.

**Table 3:** Soil Background Heavy Metals for Dry and Wet Season in the Study Area.

Heavy Metal Concentration (mg/kg)	Wet Season	Dry Season
Nickel (Ni)	7.46	4.8
Lead (Pb)	1.52	0.29
Chromium (Cr)	13.92	3.52
Zinc (Zn)	87.74	20.5

### Geo-accumulation index results

**Figure 8:**
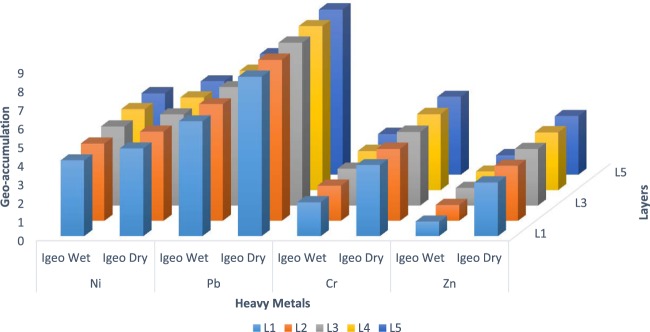
Summary of the calculated values for Geo-accumulation for Wet and Dry Season.

Figure 8 shows the computed Geo-accumulation index of the four (4) heavy metals whose their background concentration values are known. The Geo-accumulation index of the heavy metals ranged from moderately-strongly polluted to extremely-polluted in the dry season. Layer 5 had the maximum Geo-accumulation index for the heavy metals considered, and the index value decreased in this order for the heavy metals: Layer 5> Layer 4> Layer 3> Layer 2> Layer 1. The pollution from lead (Pb) in the dry season was maximum i.e. I_geo_ value> 5 for all the lithofacies considered because of their low background concentration. The pollution degree of the heavy metals in dry season decreased in this order: Pb> Ni> Cr> Zn for all the lithofacies under consideration.

For the wet season, the heavy metal pollution ranged from unpolluted-moderately for Zn to extremely-polluted for Pb. Layer 5 also had the maximum Geo-accumulation indices while Layer 1 had the lowest values with respect to the heavy metals. The pollution of Zn reduced from moderately-strongly polluted (dry season) to unpolluted-moderately polluted (wet season). The ${I}_{geo}$ values of Ni ranged from 4.06 – 4.37 (wet season) and 4.70 – 5.01 (dry season); for Pb it ranged from 6.16 – 6.47 (wet season) and 8.55 – 8.86 (dry season); for Cr, it ranged from 1.82 – 2.19 (wet season) and 3.81 – 4.18 (dry season); and for Zn, it was from 0.78 – 1.05 (wet season) and 2.88 – 3.15 (dry season). Based on the ${I}_{geo}$ values computed, Pb and Ni will have more pronounced effects than Cr and Zn. This high level of Pb may be due to upward migration of submarine sediment deposits at shallow depths in the region. These soil samples from the subsurface during drilling operations will contaminate the soils in the environment if prevention mechanisms are not put in place before drilling the hydrocarbon wells.

### Estimated contamination factor

**Figure 9:**
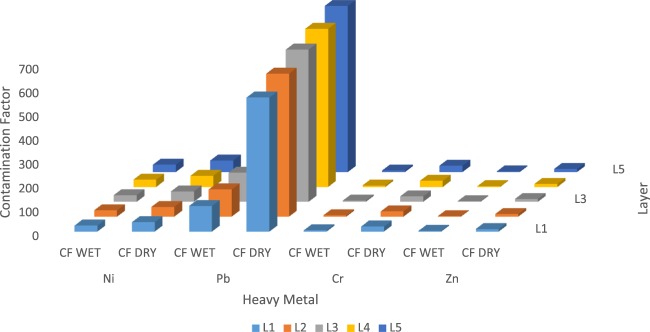
Contamination Assessment through Contamination Factor for the Lithofacies.

Figure 9 shows the computed results for each lithofacies under consideration, and the excel work sheet for the calculations is attached as supplementary document. There are mainly four classifications for expressing estimated contamination factors; CF < 1 refers to the low contamination factor, 1 ≤ CF < 3 refers to the moderate contamination factor, 3 ≤ CF < 6 refers to the considerable contamination factor, and CF ≥ 6 refers to the very high contamination factor. The contamination factor of Pb is very high (i.e. between 562 and 696) relative to the baseline study of the area. The contamination factor for the heavy metals in the lithofacies decreased in this order: Pb> Ni> Cr> Zn in the dry and wet seasons. The CF values computed for the four (4) heavy metals show that the field-environment soil will be moderately contaminated by Ni, Cr, Zn but considerably contaminated by Pb.

### Calculated ecological risk potentials

Table 4 shows the standard grading of potential ecological risks estimated for the heavy metals in different subsurface lithofacies.

**Table 4:** Heavy Metals Grading Standard of Potential Ecological Risk^28^.

${{\boldsymbol{E}}}_{{\boldsymbol{R}}}^{{\boldsymbol{i}}}$	Pollution Degree	RI	Risk level	Risk Degree
${E}_{R}^{i}$ < 30	Slight	RI < 40	A	Slight
30 ≤ ${E}_{R}^{i}$ < 60	Medium	40 ≤ RI < 80	B	Medium
60 ≤ ${E}_{R}^{i}$ < 120	Strong	80 ≤ RI < 160	C	Strong
120 ≤ ${E}_{R}^{i}$ < 240	Very strong	160 ≤ RI < 320	D	Very strong
${E}_{R}^{i}$. ≥ 240	Extremely strong	RI ≥ 320	—	—

**Figure 10:**
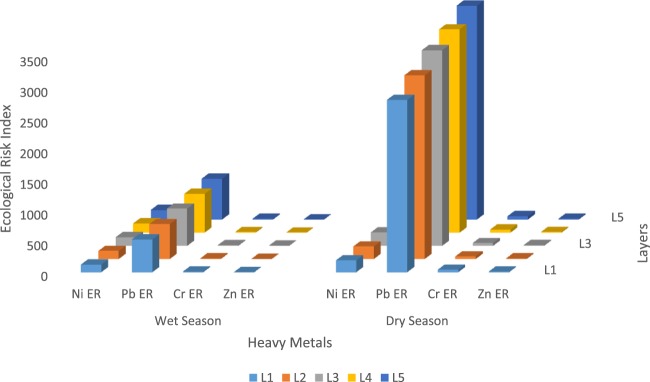
Ecological risk Index for the different Subsurface Lithofacies.

Fig. 10 and Table 5 represent the potential ecological risk indices and the comprehensive potential ecological risk indices of the heavy metals in the study area in the dry and wet seasons. Figure 10 shows that the potential ecological risk indices of the heavy metals from the different lithofacies are higher in dry season relative to the wet season. For Pb, the potential ecological risk ranges from 536 – 664 in the wet season (extremely strong degree of pollution) and 2810 – 3480 in dry season (extremely strong degree of pollution); the Cr index value ranges from 10.6 – 13.7 (slight degree of pollution) in the wet season to 41.9 – 54.2 in dry season (medium degree of pollution); the Ni value ranges from 125 − 155 (very strong degree of pollution) in wet season to 194 – 242 (extremely strong degree of pollution) in dry season; the value for Zn ranges from 2.58 – 3.10 (slight degree of pollution) in wet season to 11.1 – 13.3 (slight degree of pollution) in the dry season. Among the four (4) heavy metals considered, the degree of pollution caused by Ni and Cr during these two seasons changed significantly, but those of Pb and Zn did not change. It was also observed that the deeper subsurface lithofacies in this area of study contain high concentrations of the heavy metals^38,39^. The degree of pollution in these different lithofacies, increased from layer 1 up to layer 5. Thus, the degree of pollution from the lithofacies decreased in this order: Layer 5> Layer 4> Layer 3> layer 2> Layer 1.

**Table 5:** Comprehensive Potential Ecological Risk Levels for the Heavy Metals.

Heavy Metal	RI value	Risk Level	Risk degree	RI value	Risk Level	Risk degree
	Wet Season			Dry Season		
Cr	59.9	B	Medium	237	D	Very strong
Zn	14.3	A	Slight	61.1	A	Slight
Ni	706	—	—	1100	—	—
Pb	3010	—	—	1580	—	—

Table 5 shows the comprehensive potential ecological risk index of the heavy metals. The degree of heavy metal pollution in the ecosystem of the study area decreased in this order: Pb> Ni> Cr> Zn. The standard adopted (Table 4) did not consider higher range of RI values; thus, this present study could not classify the comprehensive potential ecological risk levels of Ni and Pb. The potential ecological risk level of Cr moved from medium risk degree in wet season to very strong risk degree during the dry season. But, the potential ecological risk level of Zn remained “slight” in the wet and dry seasons. This higher Geo-accumulation index noted at the depth in Layer 5 may be attributed to the effect of water basin with turbidity currents, deltas, and shallow marine sediment deposits with storm impacted conditions.

The study by Raulinaitis et al.^40^ on two layers of sediments reveals much higher pollution levels in the subsurface (0.6 – 1.2 m) than surface (0 – 0.6 m) level by most metals, namely As, Cd, Hg, Ni, Sb and Sn, while Zn was the only metal with higher concentrations in the surface level. They were not able to establish a distribution pattern for the most metals due to shallow depth, and identical physical and chemical properties. Literature have shown that the heavy metal distribution for gold mining area varies significantly and decrease in the order of Cr> Ni> As> Zn> Cu> Co> Pb> Hg> Cd. The average ranges were as follows: Cr (77.50–861.67 mg/kg); Ni (68.33–152.50 mg/kg); As (65.17–115.19 mg/kg); Zn (21.82–82.50 mg/kg); Cu (19.09–55.83 mg/kg); Co (11.82–33.68 mg/kg); Pb (1.58–10.22 mg/kg); Hg (0.06–0.13 mg/kg); and Cd (0.04–0.05 mg/kg) respectively^41^. Thus, showing that the soils surrounding the gold mining area are polluted by the heavy metals. Pb, Cd, Hg, Zn and Ni concentration in this present study are significantly higher than most studies in literature. The level of heavy metal contamination in the study area soil analysed using seasonal geoaccumulation index shows that the risk degree is more significant during the dry season. Thus, the lithofacies produced during the drilling operation have significant concentration of heavy metals that can affect the environmental background values, because Cr risk degree is very strong (Table 5). Also, this study established the concentration distributions of these heavy metals in the lithofacies as the depth increases downwards.

## Conclusion

This work presents a study on the heavy metal pollution analysis, health risk and potential ecological risk assessments around a hydrocarbon field during drilling operations. The present study was performed through investigation, field sampling, laboratory experiment and mathematical analysis of the generated data. Five (5) different subsurface lithofacies were considered and the conclusions can be summarized as follows:

The results of this study provide valuable information about the heavy metal contamination in the subsurface lithofacies of the hydrocarbon well considered. The average concentrations of the heavy metals present in the formation samples varied significantly and decreased in the order of Al> Zn> Ni> Pb> Cr> Cu> Cd> As> Hg. The highest concentration of Al, Cu, and Zn in this present study were within the maximum allowable limits according to US EPA (2002). But, the highest concentration of As, Cd, Hg and Ni were all above the maximum allowable limits of 20 mg/kg, 3 mg/kg, 30 mg/kg and 72 mg/kg respectively (US EPA, 2002).

The results from the potential health risk estimates, indicated that the maximum mean daily dose of Pb (9.18 × 10^−6^ mg/kg/d) and Cr (1.42 × 10^−6^ mg/kg/d) can be a concern for the drilling crew and the environment. The Geo-accumulation analysis showed that Layer 5 had the maximum Geo-accumulation index for the heavy metals considered, and the index value decreased in this order for the heavy metals: Layer 5> Layer 4> Layer 3> Layer 2> Layer 1. The pollution from lead (Pb) in the dry season was maximum (I_geo_ value> 5) for all the lithofacies considered.

The results of the potential ecological risk assessment indicate that the degree of pollution in these different lithofacies, increased from layer 1 up to layer 5. The degree of heavy metal pollution degree in the ecosystem’s study area decreased in this order: Pb> Ni> Cr> Zn. The standard adopted did not consider higher range of RI values; thus, this present study could not classify the comprehensive potential ecological risk levels of Ni and Pb. But, the potential ecological risk levels of Cr moved from “medium risk” in the wet season to “very strong risk” during the dry season; and that of Zn remained slight for the wet and dry seasons. The findings from this present study can provide useful insights for developing a precautionary strategy for oil and gas regulatory authorities in regulating the toxicity exposure risk levels of the highlighted heavy metals to drillers and people in the environment. The results are also expected to be useful in creating awareness campaigns as well as policy formulation and implementation aimed at protecting the environment and lives of drillers/operators in the oil and gas industry. Also, the results from this study will help to provide appropriate mitigation measures and Environmental Management Plans (EMPs) on season basis, since the level of impact differed within the two seasons considered in the study area.

## Supplementary information

Supplementary Information.
